# Supplementary material for: Association of sleep quality and sleep duration with serum uric acid levels in adults
Source: PLoS One. 2020 Sep 17;15(9):e0239185. doi: 10.1371/journal.pone.0239185 (PMC7497980; doi:10.1371/journal.pone.0239185)
Supplement: Attachment — Submitted filename: Response to Reviewers_0811_v2 FINAL.docx [file pone.0239185.s002.docx]

**To reviewer #1:**

Thank you very much for your valuable comments. The revised parts of the paper, including the responses to reviewers and English editing, have been highlighted in yellow for your reference. We hope the following amendments will satisfy your suggestions.

1.While the paper is well-written and the statistics appear appropriate, I am unsure of the clinical significance of the statistical finding. In particular, if you look at Table 1, the UA difference is only 0.1 mg/dL between the two groups. This is statistically difference but not sure it is clinically significant, since both levels are likely in normal range. Then, if you look at Table 3, it would appear that the differences in UA between the two groups, after various corrections, remains quite small. Thus, it is not clear to me that the statistical difference in UA has any clinical outcomes that are important.

Therefore, the paper would be greatly enhanced if the authors had any outcome data related to UA that showed that the small differences make a clinical difference.

**Reply:**

Thanks for your suggestion. We agree that the effect of sleep quality on uric acid may be relatively small. However, this minor but statistically significant finding provided a research direction for further exploring the association between sleep and uric acid. Further study is needed for clarifying this relationship and we had added this point as one limitation in DISCUSSION section of the revised manuscript. According to previous studies, high-normal level of serum uric acid was also related risk of diabetes [1], impaired renal function [2] and even mild cognitive impairment [3]. Further studies are needed to clarify their relationship and we had added this point as one limitation in DISCUSSION section of the revised manuscript.

We have added the above points in the INTRODUCTION **(Line 27)** and DISSCUSSION **(Line 253-257)** section as follows:

| Section | Original part | Revised part |
| --- | --- | --- |
| Introduction  (Line 27 , paragraph 1, page 3 of revised manuscript) | …early mortality [9]. However, low uric acid… | …early mortality [9]. In addition, some studies have demonstrated that high-normal levels of serum uric acid are also associated with risk of diabetes [10], impaired renal function [11] and even mild cognitive impairment [12]. However, low uric acid… |
| Discussion  (Line 253, paragraph 6, page 14 of revised manuscript) | …polysomnography. Forth, the sample size of long sleepers was relatively small and then there was an underestimation for the relationship between long sleep duration and uric acid level. Finally, the socioeconomic status[68]… | …polysomnography. ~~Forth~~Fourth, the sample size of long sleepers was relatively small, ~~and then there was an underestimation for~~, and the relationship between long sleep duration and uric acid levels was underestimated. Fifth, the effects of sleep quality on uric acid were relatively small, but significant, in the present study. These results provide a research direction for further studies exploring the association between sleep and uric acid to clarify their relationship. Finally, ~~the~~ socioeconomic status ~~[68]~~[75]… |

**Reference:**

1.	Shani M, Vinker S, Dinour D, Leiba M, Twig G, Holtzman EJ, et al. High Normal Uric Acid Levels Are Associated with an Increased Risk of Diabetes in Lean, Normoglycemic Healthy Women. The Journal of clinical endocrinology and metabolism. 2016;101(10):3772-8. Epub 2016/08/18. doi: 10.1210/jc.2016-2107. PubMed PMID: 27533308.

2.	Ficociello LH, Rosolowsky ET, Niewczas MA, Maselli NJ, Weinberg JM, Aschengrau A, et al. High-normal serum uric acid increases risk of early progressive renal function loss in type 1 diabetes: results of a 6-year follow-up. Diabetes care. 2010;33(6):1337-43. Epub 2010/03/25. doi: 10.2337/dc10-0227. PubMed PMID: 20332356; PubMed Central PMCID: PMCPMC2875450.

3.	Schretlen DJ, Inscore AB, Jinnah HA, Rao V, Gordon B, Pearlson GD. Serum uric acid and cognitive function in community-dwelling older adults. Neuropsychology. 2007;21(1):136-40. Epub 2007/01/05. doi: 10.1037/0894-4105.21.1.136. PubMed PMID: 17201536.

**To reviewer #2:**

Thank you very much for your valuable comments. The revised parts of the paper, including the responses to reviewers and English editing, have been highlighted in yellow for your reference. We hope the following amendments will satisfy your suggestions.

1. This article is a report of regression on a large dataset exploring the relationship between uric acid levels and duration of sleep. This article will contribute meaningful evidence to the scientific literature. It appears to be scientifically developed and a good discussion of the reasons for the relationship are discussed.

I am recommending that the authors consider adding one variable to their dataset that could be a mediator to the relationship. The authors will also need to edit the grammar and sentence structure in below mentioned sections of the paper.

Introduction:

Line 45: typo “lover”

Line 47: poor grammar/sentence structure

There is no mention of the possible physiological relationship between sleep duration and uric acid levels. This must be delineated to substantiate the scientific underpinnings of the study.

**Reply:**

Thanks for your reminding. We had already corrected the typing error in INTRODUCTION section. Besides, we carefully rechecked the sentence in line 47 and found that the result of one study (from Kanagasabai et al., reference 42) was similar to another study (from Papandreou et al., reference 41). The other study in that sentence (from Wiener et al., reference 44) showed a different result. Thus, we re-organized this part and the English editing was done by professional editor. In addition, we also added possible physiological relationship between sleep duration and uric acid levels in the INTRODUCTION section.

We have added the above points in the INTRODUCTION **(Line 40-44, Line 47-51)** section as below:

| Section | Original part | Revised part |
| --- | --- | --- |
| Introduction  (Line 40, paragraph 3, page 3 of revised manuscript) | As for the relationship between sleep-related conditions and uric acid, most previous studies found that sleep-disordered breathing such as obstructive sleep apnea (OSA) was associated with hyperuricemia and gout[29-32]. However, studies investigating the relationship between sleep quality or sleep duration and uric acid levels are limited[33]. | ~~As for the relationship between~~ Physiologically, sleep ~~related conditions~~ mediates the level of catecholamine and cortisol [32, 33], which may potentially influence uric acid levels [34]. Most previous studies have found that sleep-disordered breathing such as obstructive sleep apnea (OSA) ~~was~~is associated with hyperuricemia and gout ~~[29-32]~~[35-38]. However, studies investigating the relationship between sleep quality or sleep duration and uric acid levels are limited ~~[33]~~[39-44]. |
| Introduction  (Line 47, paragraph 3 , page 4 of revised manuscript) | As for the relationship between sleep duration and uric acids, one demonstrated that sleep duration was inversely associated with serum uric acids in the elderly people[36], and another study found that long sleepers hand lover prevalence of hyperuricemia[37]. However, two other studies failed to determine an association between sleep duration and uric acid level [38, 39]. | As for the relationship between sleep duration and uric acid~~s~~, ~~one~~two studies demonstrated that sleep duration was inversely associated with serum uric acids ~~in the elderly people[36]~~[41, 42], and another study found that long sleepers ~~hand lover~~ had a lower prevalence of hyperuricemia ~~[37]~~[43]. However, ~~two other studies failed to determine an association~~ the relationship between sleep duration and uric acid level [38, 39]~~.~~was insignificant in another study based on the National Health and Nutrition Examination Survey (NHANES) database [44]. |

2. Study Population:

Poor grammar and sentence structure.

Why did you exclude medications? Again, poor writing.

**Reply:**

Thanks for your comment. According to previous studies, some medication for treating hypertension, dyslipidemia and diabetes influenced serum uric acid level directly [4-6]. For example, atorvastatin, one commonly used lipid-lowering drug, and pioglitazone, an anti-diabetic agent, both lowers serum uric acid levels [4, 6]. As for antihypertensive drugs, calcium-channel blockers and lorsartan lowers risk of gout while diuretics and beta-blockers increased the risk of gout [5].Therefore, based on avoiding the potential confounding effect of these medication on uric acid levels, we excluded subjects who had used such medication. Besides, the English editing was done by professional editor.

We have added the re-edited version of “study population” in the METHOD section as below **(Line 58-64).** (We also modified the whole article under professional proofreader’s suggestion. All other the changes can be seen in both “Manuscript” and “Revised Manuscript with Track Changes”, which were uploaded in the online submission system.)

| Section | Original part | Revised part |
| --- | --- | --- |
| METHOD  (Line 58, paragraph 1, page 4 of revised manuscript) | A total of 4,592 patients who visited for self-motivated general health assessment were recruited in this study, and data were obtained from a health examination center in National Cheng Kung University Hospital, from October 2001 to August 2009. This study used a secondary data without any personal identifiable information and it was approved by the Institutional Review Board of NCKUH in Taiwan (IRB number: A-ER-107-285). Participants aged <18 years, medications for hyperuricemia, hypertension, DM or dyslipidemia, and incomplete data were excluded. | A total of 4,592 patients who visited a health center for a self-motivated general health assessment were recruited in this study~~,and data~~ Data were obtained from a health examination center ~~in~~at National Cheng Kung University Hospital, from October 2001 to August 2009. ~~This study used a secondary~~ Secondary data without any personal identifiable information was used, and ~~it~~the study was approved by the Institutional Review Board of NCKUH in Taiwan (IRB number: A-ER-107-285). Participants aged <18 years, on medications for hyperuricemia, hypertension, DM or dyslipidemia, and those with incomplete data were excluded. |

**Reference:**

4.	Milionis HJ, Kakafika AI, Tsouli SG, Athyros VG, Bairaktari ET, Seferiadis KI, et al. Effects of statin treatment on uric acid homeostasis in patients with primary hyperlipidemia. American heart journal. 2004;148(4):635-40. Epub 2004/10/02. doi: 10.1016/j.ahj.2004.04.005. PubMed PMID: 15459594.

5.	Choi HK, Soriano LC, Zhang Y, Rodríguez LA. Antihypertensive drugs and risk of incident gout among patients with hypertension: population based case-control study. BMJ (Clinical research ed). 2012;344:d8190. Epub 2012/01/14. doi: 10.1136/bmj.d8190. PubMed PMID: 22240117;

6.	Niu SW, Chang KT, Lin HY, Kuo IC, Chang YH, Chen YH, et al. Decreased incidence of gout in diabetic patients using pioglitazone. Rheumatology (Oxford). 2018;57(1):92-9. Epub 2017/10/19. doi: 10.1093/rheumatology/kex363. PubMed PMID: 29040733.

3. Clinical measurements:

There is no mention of the validity and reliability of the PSQI sleep duration question. What construct is the sleep duration question really accessing? Is subjective report of sleep a valid measure? This must be substantiated.

**Reply:**

Thanks for your suggestion. According to previous studies, the Chinese version of the PSQI was an validated questionnaire for evaluating multiple dimensions of sleep (including both sleep quality and sleep duration) by 7 components: habitual sleep efficiency, sleep latency, sleep duration, subjective sleep quality, use of sleep medications, sleep disturbances, and presence of daytime dysfunction. The Chinese version of the PSQI and had an overall reliability coefficient of 0.82-0.83 and good test-retest reliability with a coefficient of 0.85. A Chinese version of PSQI global score > 5 had a diagnostic sensitivity of 98% and specificity of 55% in distinguishing poor and good sleepers.[7]. However, despite with acceptable validity and reliability, PSQI may have its limitation in evaluating sleep only by self-reported questionnaire, comparing to other objective measurements such polysomnography (PSG) or sleep actigraphy. Since PSG or sleep actigraphy were not available in this study, we had mentioned this limitation in the section of DISCUSSION.

We have added the above points in the METHOD **(Line 72-84)** and DISCUSSION **(Line 251-253)** section as below.

| Section | Original part | Revised part |
| --- | --- | --- |
| METHOD  (Line 72, paragraph 2, page 5 of revised manuscript) | …times per week. Sleep duration and quality was assessed using the Chinese version of the PSQI[40], which evaluated participants’ sleep condition over a 1-month time interval using a self-rated questionnaire. The PSQI demonstrates problems in sleep duration, habitual sleep efficiency, sleep latency, subjective sleep quality, use of sleep medications, sleep disturbances, and presence of daytime dysfunction. The total global PSQI scores range from 0 to 21 points, and a lower global PSQI score indicates better sleep quality. Participants with a global PSQI score of >5 is defined as a poor sleeper. Sleep duration was categorized into three groups: short (<7 h/day), normal (7–9 h/day), and long (≧9 h/day)[41]. | …times per week. Sleep duration and quality ~~was~~were assessed using the Chinese version of the PSQI ~~[40]~~ [45], which ~~evaluated~~ was used to evaluate the participants’ sleep ~~condition~~ quality over a 1-month time interval using a self-rated questionnaire. The Chinese version of the PSQI ~~demonstrates problems in~~ was validated as an assessment tool for evaluating sleep condition, with an overall reliability coefficient of 0.82-0.83, and good test-retest reliability, with a coefficient of 0.85. A Chinese version of the PSQI global score > 5 had a diagnostic sensitivity of 98% and specificity of 55% in terms of distinguishing poor and good sleepers [45]. The PSQI reflects seven important aspect of sleep, including sleep duration, habitual sleep efficiency, sleep latency, subjective sleep quality, use of sleep medications, sleep disturbances, and presence of daytime dysfunction. The total global PSQI scores range from 0 to 21 points, ~~and~~ where a lower global PSQI score indicates better sleep quality. Participants with a global PSQI score of >5 ~~is~~were defined as ~~a~~poor sleepers. Sleep duration was categorized into three groups: short (<7 h/day), normal (7–9 h/day), and long (≧9 h/day)~~[41]~~[46]. |
| DISCUSSION  (Line 251, paragraph 6, page 14 of revised manuscript) | Third, our participants’ sleep quality and duration were obtained from self-reported questionnaires but not measured by objective medical devices, such as polysomnography. | Third, our participants’ sleep quality and duration were obtained from self-reported questionnaires but not measured ~~by~~using objective medical devices, such as polysomnography or sleep actigraphy. |

**Reference:**

7.	Tsai PS, Wang SY, Wang MY, Su CT, Yang TT, Huang CJ, et al. Psychometric evaluation of the Chinese version of the Pittsburgh Sleep Quality Index (CPSQI) in primary insomnia and control subjects. Quality of life research : an international journal of quality of life aspects of treatment, care and rehabilitation. 2005;14(8):1943-52. Epub 2005/09/13. doi: 10.1007/s11136-005-4346-x. PubMed PMID: 16155782.

4. Clinical measurements:

Validation and measurement specifications and moderators of the uric acid test are not discussed. For example, does diet the day of the test have an impact on the uric acid level? How about time of day the blood was drawn? Was there only one data point for the uric acid level?

**Reply:**

Thanks for your suggestion. In our study, there was only one data point for the UA level and all the blood samples were drawn from 7 am to 9 am in the morning after the participants were under fasting condition for at least 12 hours to diminish the potential confounding effect of physiological fluctuation and dietary factors on uric acid levels[8-11].

We have added the above points in the METHOD section as below **(Line 92-94).**

| Section | Original part | Revised part |
| --- | --- | --- |
| METHOD  (Line 92 , paragraph 4, page 5 of revised manuscript) | After fasting for at least 12 h, all participants underwent blood sampling for basic biochemical examinations. None of the… | After fasting for at least 12 h, all participants underwent blood sampling for basic biochemical examinations. All of the blood samples were drawn from 7 am to 9 am in the morning. None of the… |

**Reference:**

8.	Maclachlan MJ, Rodnan GP. Effect of food, fast and alcohol on serum uric acid and acute attacks of gout. The American journal of medicine. 1967;42(1):38-57. Epub 1967/01/01. doi: 10.1016/0002-9343(67)90005-8. PubMed PMID: 6016479.

9.	Dalbeth N, Wong S, Gamble GD, Horne A, Mason B, Pool B, et al. Acute effect of milk on serum urate concentrations: a randomised controlled crossover trial. Annals of the rheumatic diseases. 2010;69(9):1677-82. Epub 2010/05/18. doi: 10.1136/ard.2009.124230. PubMed PMID: 20472590.

10.	Carran EL, White SJ, Reynolds AN, Haszard JJ, Venn BJ. Acute effect of fructose intake from sugar-sweetened beverages on plasma uric acid: a randomised controlled trial. European journal of clinical nutrition. 2016;70(9):1034-8. Epub 2016/06/23. doi: 10.1038/ejcn.2016.112. PubMed PMID: 27329612.

11.	Devgun MS, Dhillon HS. Importance of diurnal variations on clinical value and interpretation of serum urate measurements. Journal of clinical pathology. 1992;45(2):110-3. Epub 1992/02/01. doi: 10.1136/jcp.45.2.110. PubMed PMID: 1541689; PubMed Central PMCID: PMCPMC495647.

5. Results and Analysis:

Ok, although the PSQI is a valid screen for obstructive sleep apnea. Why didn’t you look at the relationship between OSA and uric acid levels? Do you have any measures of inflammation in your dataset? If so, you might want to look at that relationship.

**Reply:**

Thanks for your reminding. OSA has been shown to be strongly related to hyperuricemia and gout. However, diagnostic instrument for OSA such as polysomnography (PSG) was not available in this study. Hence, we could only exclude subjects with OSA by self-reported history to reduce its confounding effect, and thus may have underestimated its influence. This limitation was added in the DISCUSSION section. **(Line 248-251)**

As for systemic inflammation markers and uric acid level, we had added white blood cell (WBC) count in the multivariate analysis **(see** **Line 96-97、Line 115-116 of the revised manuscript).** In univariate analysis **(Line 123)**, poor sleepers had higher WBC count than good sleepers **(Table 1)**. However, the WBC count was not significantly different among subjects with different sleep duration **(Table 2)**. Multiple regression model **(Line 141-148, Table 3)** demonstrated that, after adjustment of covariates such as age, gender, obesity, hypertension, diabetes, dyslipidemia, WBC count, eGFR, alcohol consumption, smoking and exercise, poor sleep quality was still significantly related to lower uric acid level (b coefficient: -0.085, confidence interval (CI): −0.161 to −0.008, p = 0.030). Besides, short sleep duration was associated with higher serum uric acid levels (b-coefficient: 0.106, 95% CI: 0.031–0.181, p = 0.006). However, no association was found between long sleeper duration and uric acid levels. The relationship between sleep quality, sleep duration and uric acid level was similar to our original analysis, and the b-coefficient of poor sleep quality and short sleep duration increased slightly when WBC count was taken into consideration. In addition, WBC count was positively related to uric acid levels.

We have added the above points in the METHOD **(Line 96-97、Line 115-116)**, RESULT **(Line 123, Line 141-148)** and DISCUSSION **(Line 180-182、Line 222-223, Line 227-231, Line 248-251)** section as below. We also modified the content of Table 1 to table 3 due to adding WBC count into statistical analysis.

| Section | Original part | Revised part |
| --- | --- | --- |
| Methods  (Line 96, paragraph 3, page 5 of revised manuscript) | …alanine aminotransferase (ALT), and creatinine levels were collected. | …alanine aminotransferase (ALT), white blood cell (WBC) count, and creatinine levels were collected. |
| Methods  (Line 115, paragraph 3, page 5 of revised manuscript) | …obesity, estimated glomerular filtration rate (eGFR) <60, TC/HDL-C ratio, DM, HTN, current smoking, alcohol consumption… | …obesity, estimated glomerular filtration rate (eGFR) <60, TC/HDL-C ratio, WBC count, DM, HTN, current smoking, alcohol consumption… |
| Result  (Line 123, paragraph 1, page 7 of revised manuscript) | …blood pressure, uric acid, FPG, TC, and renal functions. | …blood pressure, uric acid, FPG, TC, WBC count, and renal functions. |
| Result  (Line 141, paragraph 2 , page of revised manuscript) | As seen in Table 3, when adjusted for sleep duration, poor sleepers remained associated with lower uric acid levels [b-coefficient: −0.078, 95% confidence interval (CI): −0.155 to −0.002, p = 0.044] and short sleep duration was associated with higher serum uric acid levels (b-coefficient: 0.101, 95% CI: 0.026–0.177, p = 0.009). However, no association was found between long sleeper duration and uric acid levels. In addition, uric acid levels were positively associated with overweight and obesity, TC/HDL-C ratio, hypertension, and alcohol consumption habits, but decreased eGFR levels. | As ~~seen~~shown in Table 3, when adjusted for sleep duration, poor sleepers remained associated with lower uric acid levels [b-coefficient: ~~−0.078~~−0.085, 95% confidence interval (CI): ~~−0.155~~−0.161 to ~~−0.002~~−0.008, p = ~~0.044~~ 0.030], and short sleep duration was associated with higher serum uric acid levels (b-coefficient: ~~0.101~~ 0.106, 95% CI: ~~0.026~~ 0.031– ~~0.177~~ 0.181, p = ~~0.009~~ 0.006). However, no association was found between long sleeper duration and uric acid levels. In addition, uric acid levels were positively associated with overweight and obesity, the TC/HDL-C ratio, WBC count, hypertension, and alcohol consumption habits, but decreased eGFR levels. |
| Discussion  (Line 180, paragraph 2, page 12 of revised manuscript) | The possible mechanism between sleep quality and uric acid remains unknown. The beneficial aspect of uric acid may play a role among good sleepers. | The possible mechanism for the relationship between sleep quality and uric acid remains ~~unknown~~ unclear. ~~The~~ Since sleep disturbances are related to increased systemic inflammation and oxidative stress [32], the beneficial aspects of uric acid may play a role among good sleepers. |
| Discussion  (Line 222, paragraph , page of revised manuscript) | hypertension, lipid profile, eGFR, and alcohol consumption, and the results were consistent with that of previous studies[58, 59]. | …hypertension, lipid profile, decreased eGFR, WBC count and alcohol consumption. ~~and the~~ These results were consistent with ~~that~~ those of previous studies ~~[58-59]~~[62, 63]. |
| Discussion  (Line 227, paragraph 5, page 14 of revised manuscript) | …increased urate production [61, 62]. As for the negative… | …increased urate production ~~[61, 62]~~ [65, 66]. Also, obesity is related to increased visceral fat accumulation, which may result in increased plasma free fatty acids in hepatic tissue and subsequently lead to elevated uric acid production [67]. Higher WBC counts provoke production of tumor necrosis factor-alpha and interleukin-6, which may lead to impaired insulin sensitivity and in turn to higher uric acid levels [68, 69]. As for the negative… |
| Discussion  (Line 248, paragraph 6, page 14 of revised manuscript) | Second, participants with OSA, which was associated with hyperuricemia and gout[29, 31], was excluded only by self-reported history and thus may have underestimated its influence. | Second, participants with OSA, which is associated with hyperuricemia and gout ~~[29, 31]~~ [35, 37], was excluded only by self-reported history, due to the unavailability of subjective measurements such as polysomnography, and ~~thus~~ therefore, its influence may have been underestimated ~~its influence~~. |

6. Results and Analysis:

Really nice review of the literature and discussion, but I think you are missing the point about OSA/obesity/uric acid levels.

**Reply:**

Thanks for your reminding. As mentioned above, since diagnostic instrument for OSA such as polysomnography was not available in this study, we could only exclude those with past history of OSA for alleviating the confounding effect of OSA. As for obesity and uric acid, we agreed that obesity plays an important role in development of hyperuricemia, and thus we have added additional statement in the DISCUSSION section as below **(Line 227-231, Line 248-251)**

| Section | Original part | Revised part |
| --- | --- | --- |
| Discussion  (Line 227, paragraph 5, page 14 of revised manuscript) | …increased urate production [61, 62]. As for the negative… | …increased urate production ~~[61, 62]~~ [65, 66]. Also, obesity is related to increased visceral fat accumulation, which may result in increased plasma free fatty acids in hepatic tissue and subsequently lead to elevated uric acid production [67]. Higher WBC counts provoke production of tumor necrosis factor-alpha and interleukin-6, which may lead to impaired insulin sensitivity and in turn to higher uric acid levels [68, 69]. As for the negative… |
| Discussion  (Line 248, paragraph 6, page 14 of revised manuscript) | Second, participants with OSA, which was associated with hyperuricemia and gout[29, 31], was excluded only by self-reported history and thus may have underestimated its influence. | Second, participants with OSA, which is associated with hyperuricemia and gout ~~[29, 31]~~ [35, 37], was excluded only by self-reported history, due to the unavailability of subjective measurements such as polysomnography, and ~~thus~~ therefore, its influence may have been underestimated ~~its influence~~. |
